# Metals and trace element concentrations in breast milk of first time healthy mothers: a biological monitoring study

**DOI:** 10.1186/1476-069X-11-92

**Published:** 2012-12-14

**Authors:** Karin Ljung Björklund, Marie Vahter, Brita Palm, Margaretha Grandér, Sanna Lignell, Marika Berglund

**Affiliations:** 1 Institute of Environmental Medicine, Karolinska Institutet, PO Box 210, 171 77, Stockholm, SE, Sweden; 2 National Food Agency, PO Box 622, 751 26, Uppsala, SE, Sweden

**Keywords:** Breast milk, Toxic metals, Trace elements, Infant exposure, Microelements, Macroelements

## Abstract

**Background:**

Breast milk is the best source of nutrition for the newborn infant. However, since all infants cannot be breast-fed, there is a need for background data for setting adequate daily intakes. Previously, concentration data on major essential elements and some toxic elements in breast milk, based on different analytical techniques, have been published. There is no recent study on a large number of metals and trace elements in breast milk, using a sensitive analytical method for determination of low element concentrations.

**Methods:**

Breast milk concentrations of 32 metals and elements in early lactation (days 14-21) were determined in a random sample of first time Swedish mothers (n = 60) using inductively coupled plasma mass spectrometry (ICPMS).

**Results:**

There were small inter-individual concentration variations in the macroelements Ca, K, Mg, P and S, and striking similarities across studies and over time, supporting a tight regulation of these elements in breast milk. Large inter-individual and over time differences were detected for Na concentrations, which may reflect an increase in salt consumption in Swedish women. Large inter-individual differences were also detected for the microelements Co, Cr, Mn and Mo, and the toxic metals As, Cd, Pb, Sb and V. Arsenic and B were positively correlated with fish consumption, indicating influence of maternal intake on breast milk concentrations. Observed differences in breast milk element concentrations across studies and over time could be attributed to the timing of sampling and a general decline over time of lactation (Cu, Fe, Mo, Zn), a possible lack of regulation of certain elements in breast milk (As, B, Co, Mn, Se) and time trends in environmental exposure (Pb), or in some cases to differences in analytical performance (Cr, Fe).

**Conclusions:**

This study provides reliable updated information on a number of metals and elements in breast milk, of which some have not previously been reported.

## Background

Breast milk is the best source of nutrition for the newborn infant, and exclusive breast feeding is recommended up to 6 months of age [1]. In general, breast milk contains all nutrients required for proper infant development [2]. Since all infants cannot be breast-fed, the composition of infant formulas is constantly developed in order to provide the same nutrition as breast milk. Recently it was estimated that formula fed Swedish infants receive markedly higher intakes of both essential elements and toxic metals, compared to estimated intakes via breast feeding [3]. Unfortunately, the only breast milk data available for comparison was from a study published jointly by WHO and IAEA in 1989, and which is still largely used as background data for setting adequate daily intakes for infants for many elements [4]. However, analytical methods at the time did not allow for measuring low element concentrations with certainty, and the WHO/IAEA report particularly points out the difficulties with analyzing certain trace elements in biological materials [4].

Concentration data on some toxic elements and major essential elements in breast milk in women from different countries, based on different analytical techniques, have been published since then [5-11]. There is however, no recent study on a large number of both essential and toxic elements in breast milk, particularly not from European women. Furthermore, it is not clear how the major changes in dietary patterns over the last decades have influenced breast milk content of trace elements or metal contaminants.

The current study aims to provide updated information on concentrations of a wide range of toxic and essential elements in breast milk collected from Swedish women, using a sensitive analytical method for determination of low element concentrations. The results are compared with those of Swedish women in the WHO study from 1989, and also with more recent concentration data on breast milk, based on modern, sensitive analytical techniques, from other parts of the world.

## Methods

### Study population

Breast milk samples were obtained from an ongoing national trend study of environmental pollutants exposure conducted in Sweden [12]. First-time mothers who delivered at Uppsala University Hospital were randomly recruited and invited to participate in the study by donating breast milk samples. Mothers giving birth during the first week of the month and on different days according to a randomization protocol were asked to participate. Milk samples were available from the years 2000-2002 (n =30) and 2009 (n = 30), and were included in the study. Data on maternal age, weight, and fish consumption were obtained via interviews and questionnaires within the national trend study, as reported previously [13].

### Sampling

The aim was to sample 500 mL milk from each mother during 7 days of sampling. The mothers sampled milk at home during the third week after delivery (days 14-21 postpartum). Milk was sampled using a manual breast milk pump and/or a passive breast milk sampler. The women were instructed to sample milk both at the beginning and at the end of the breastfeeding session. During the sampling week, breast milk was stored in acetone-washed bottles in freezers of the mothers’ homes. Newly sampled milk was poured on top of the frozen milk. At the end of the sampling week, a nurse visited the mother to collect the bottles. The study was approved by the Ethical Committee of the Medical Faculty at Uppsala University (Dnr 96-114). Informed consent was obtained from the participating women.

### Analysis

Breast milk samples were thawed to room temperature on a shaker and thoroughly homogenized by vigorous shaking. After thorough mixing, ~1 g of each sample were mixed with 2 mL nitric acid (65% suprapur, Merck, Darmstadt, Germany) and 3 mL deionized water, and thereafter heated at 250° C for 30 min in a Milestone ultraCLAVE II microwave digestion system (EMLS, Leutkirch, Germany). Digested samples (completely clear, colorless, and homogenous solutions) were transferred to acid-washed polyethylene tubes (SARSTEDT, Nümbrecht, Germany) and diluted with deionized water to a nitric acid concentration of 20%.

The obtained solutions were measured for 32 elements using inductively coupled plasma mass spectrometry (ICPMS; Agilent 7700x, Agilent Technologies,Tokyo, Japan) with a collision/reaction cell system, in order to minimize the influence of possible interferences, ICPMS autosampler (ASX-500 series; Agilent Technologies, USA), and a quartz MicroMist nebulizer. Standard solutions for the external calibrations (ICP Multi Element Standard Solution VI CertiPUR; Merck, Darmstadt, Germany) were prepared freshly before every run in 20% nitric acid (suprapur, Merck, Darmstadt, Germany).

Aluminum (27 Al), arsenic (75 As), cadmium (111 Cd), chromium (52 Cr), cobalt (59 Co), copper (65 Cu), iron (56 Fe), magnesium (24 Mg), manganese (55 Mn), molybdenum (95 Mo), nickel (60 Ni), phosphorous (31 P), potassium (39 K), rubidium (85 Rb), sodium (23 Na), strontium (88 Sr), sulphur (34 S), vanadium (51 V) and zinc (66 Zn) were measured in helium mode, calcium (40 Ca) and selenium (78 Se) in hydrogen mode, and antimony (121 Sb), barium (137 Ba), beryllium (9 Be), boron (11 B), cesium (133 Cs), lead (208 Pb), lithium (7 Li), silver (107 Ag), thorium (232 Th), tin (118 Sn) and uranium (238 U) in standard mode.

Internal standards, 72Ge (Li, Be, B, Na, Mg, Al, P, S, K, Ca, V, Cr, Mn, Fe, Co, Ni, Cu, Zn, As, Se), 103Rh (Mo, Cd, Sb), and 193Ir (Rb, Sr, Ag, Sn, Cs, Ba, Pb, Th, U) were used.

Speciation of arsenic in breast milk samples with highest total arsenic concentrations (2-4 μg As/L; n = 3) was performed according to Fangstrom et al. [14]. A density of 1.03 Kg/L has been reported for breast milk [15], which is in accordance with our measurements in the third week of lactation of 1.01 Kg/L, Thus, we approximate mass with volume when presenting our element concentrations and comparing with literature data.

### Quality control

Analytical quality control included sampling material chemical blanks and reference materials. In order to evaluate contamination from the bottles used for storing the milk (250 mL PYREX), three empty bottles were filled with 100 mL deionized water and placed in −20°C freezers for a week. The thawed water was then included in the analysis. The bottle blanks held <0.1% of the average concentrations of the analyzed samples for all elements except for Mo (0.5%) and U (0.2%).

No suitable reference material for human milk was available. Instead, certified reference materials included 1) Infant/Adult Nutritional Formula, NIST 1849 (reference values for Ca, Cr, Cu, Fe, K, Mg, Mn, Mo, Na, P, Se, Zn), 2) Toxic metals in bovine blood, Level 1, NIST SRM 966 (reference values for Cd and Pb), 3) Seronorm^TM^ Trace Elements Whole Blood L-1, REF 201505, LOT MR4206 (reference values for all 32 elements analyzed) and 4) Seronorm^TM^ Trace Elements Whole Blood L-2, REF 201605, LOT 0503109 (reference values for all 32 elements analyzed). The NIST materials were purchased from the National Institute of Standards and Technology, Gaithersburg, MD and the Seronorm^TM^ materials from SERO AS, Billingstad, Norway. All sample preparation and analyses were carried out at the Unit of Metals and Health, Institute of Environmental Medicine, Karolinska Institutet, Sweden.

The limit of detection (LOD) was set at 3 times the standard deviation (SD) of the mean blank values for each element, based on 5 blank samples which were included in the analytical run.

### Statistics

Statistical analyses were performed using SPSS (PASW Statistics 18). Differences in element concentrations between sampling years were tested by Mann-Whitney U test. The Shapiro-Wilk test was used to test element concentration distributions for normality. Inter-individual variations in element concentrations were evaluated based on the coefficient of variation (CV; SD/mean). Correlations between elements were assessed by Spearman correlation (rs) test.

## Results

The women included in the present study were on average 29.4 ±4.0 years of age (median 29 years) at the time of sampling and had an average BMI of 23.2 ±3.7 kg/m^2^ (median 23 kg/m^2^) prior to pregnancy.

The analytical results for all 32 elements in collected breast-milk samples are presented in Table 1. Reliable analytical results (within ±20% of reference values) were achieved for all elements in the concentration ranges of the collected samples, except for Al, Ni, Sn and Th, which were excluded from further analyses. Besides Ni, only Be concentrations were frequently below detection limit (50% of samples were below the LOD of 0.5 ng/L).

**Table 1:** Concentrations of elements (n = 32; arithmetic mean, ±standard deviation, median, maximum and minimum values) analyzed in breast milk (n = 60) collected in 2002-2009 from Swedish mothers at 2-3 weeks postpartum, limit of detection (LOD), % of samples below LOD and inter-individual variation (Coefficient of variation; CV)

**Element**	**LOD**^**a**^	**% < LOD**	**Mean**	**±SD**	**Median**	**Min**	**Max**	**CV**
Ag (μg/L)	0.0010		0.080	0.098	0.046	0.016	0.70	1.22
Al (μg/L)	12		185	584	76	21	4393	3.2
As (μg/L)	0.0070		0.55	0.70	0.33	0.041	4.6	1.3
B (μg/L)	0.26		24	10	22	8.2	63	0.44
Ba (μg/L)	0.015		12	1.8	12	8.8	22	0.15
Be (μg/L)	0.00048	50	0.002	0.007	0.002	n.d.	0.022	2.9
Ca (mg/L)	4.6		305	45	307	196	416	0.15
Cd (μg/L)	0.0027		0.086	0.045	0.075	0.028	0.27	0.52
Co (μg/L)	0.0018		0.059	0.050	0.047	0.022	0.38	0.85
Cr (μg/L)	0.10	15	0.30	0.27	0.23	0.026	1.6	0.89
Cs (μg/L)	0.00028		2.8	1.6	2.5	1.1	11	0.57
Cu (μg/L)	0.055		471	75	471	327	670	0.16
Fe (μg/L)	1.6		339	134	309	135	794	0.39
K (mg/L)	12		633	40	636	549	729	0.06
Li (μg/L)	0.034		1.4	0.32	1.4	0.79	2.8	0.23
Mg (mg/L)	0.0028		28	4.8	28	21	43	0.17
Mn (μg/L)	0.010		3.0	1.4	2.6	0.79	8.4	0.48
Mo (μg/L)	0.0025		3.5	2.7	2.4	0.80	12	0.80
Na (mg/L	0.0050		217	77	192	136	480	0.36
Ni (μg/L)	0.085	75	0.96	6.5	<0	n.d.	47	6.8
P (mg/L)	0.023		172	23	171	126	233	0.13
Pb (μg/L)	0.17		1.5	0.90	1.2	0.74	6.4	0.62
Rb (μg/L)	0.044		714	108	701	484	1038	0.15
S (mg/L)	0.17		158	20	156	118	209	0.13
Sb (μg/L)	0.0013		0.042	0.029	0.031	0.018	0.15	0.69
Se (μg/L)	0.0055		13	2.6	12	8.8	18	0.20
Sn (μg/L)	0.24	3	0.40	0.099	0.38	0.21	0.77	0.25
Sr (μg/L)	0.018		33	12	31	15	79	0.35
Th (μg/L)	0.0006		0.020	0.006	0.019	0.009	0.042	0.28
U (μg/L)	0.0009		0.42	0.40	0.30	0.0097	2.0	0.97
V (μg/L)	0.0031		0.050	0.069	0.039	0.015	0.56	1.4
Zn (μg/L)	0.086		3471	979	3524	1238	5710	0.28

^a^LOD: 3*SD of mean of chemical blanks.

Below, the elements are presented in groups of macroelements (Ca, K, Mg, Na, P, S), microelements (Co, Cr, Cu, Fe, Mn, Mo, Se, Zn) and potentially toxic elements (Ag, As, B, Ba, Cd, Cs, Li, Pb, Rb, Sb, Sr, U, V).

### Macroelements (Ca, K, Mg, Na, P, S)

Overall, concentrations of the macroelements were normally distributed except for Mg and Na. There were small inter-individual variations in element concentrations (CV ≤ 0.17) except for Na (CV = 0.36; Table 1). There were no statistically significant differences in element concentrations between the two sample collection periods 2000-2002 (n = 30) and 2009 (n = 30), except for Mg which was slightly lower in 2009 (median 27 μg/L) than in 2000-2002 (30 μg/L; p < 0.05).

### Microelements (Co, Cr, Cu, Fe, Mn, Mo, Se, Zn)

Unlike the macroelements, microelement concentrations were not normally distributed, except for Cu and Zn. The concentrations of most microelements varied more between women (CV between 0.28 and 0.89) except for Cu (CV = 0.16) and Se (CV = 0.20) (Table 1). There were no significant differences in element concentrations between the two sampling periods.

### Potentially toxic elements (Ag, As, B, Ba, Cd, Cs, Li, Pb, Rb, Sb, Sr, U, V)

Of the potentially toxic elements, only Rb concentrations were normally distributed. The inter-individual variation in concentrations between women ranged from CV = 0.15 (Ba) to CV = 1.4 (V; Table 1). There were significant differences in element concentrations in milk between the two sampling periods for B with slightly higher concentrations in 2009 (median 25 μg/L) than in 2000-2002 (20 μg/L; p < 0.05), and for V and Pb which showed slightly lower concentrations in 2009 (median 0.034 and 1.2 μg/L, respectively) compared to 2000-2002 (0.046 and 1.1 μg/L, respectively; p < 0.05).

Speciation of As in breast milk showed no content of inorganic As or its methylated metabolites.

### Correlations

Most macroelements were significantly positively correlated with each other in breast milk (rs 0.3-0.5; p < 0.01). Sodium was positively correlated with Mg (rs = 0.44, p < 0.01) and S (rs = 0.45, p < 0.01) and negatively correlated with P (rs = − 0.39, p < 0.01). Of the microelements, Fe and Se were significantly correlated (rs = 0.56; p < 0.01), and both were correlated with the macroelements (rs 0.3-0.7; p < 0.01). There was a significant negative correlation between the toxic element Cd and Ca (rs = −0.25; p < 0.05). For more information on correlations the reader is referred to Additional file 1.

Arsenic concentrations in breast milk was significantly correlated with fish consumption (times/months) (rs = 0.37; p = 0.005; n = 54). The only other element that was correlated with fish consumption was B (rs = 0.33; p = 0.01; n = 54).

### Comparisons with earlier measurements

The element concentrations in the breast milk samples (n = 60) collected in lactation week 2-3 from women in Uppsala, central Sweden (2000-2009) were compared to the concentrations measured in breast milk samples collected at 3 months postpartum (pp) in women from urban and rural areas in the south and central parts of Sweden (n = 64), reported in 1989 [4]. Higher median concentration for Na, Cu, Mo and Zn (2-6 times higher) were seen in 2000-2009 compared to 1989 data, while lower median concentrations were seen for Co, Cr, As, Pb and V (10-60% of 1989 data), and in particular for Sb (1% of 1989 data) (Table 2).

**Table 2:** **Comparison of element concentrations (median, minimum and maximum values) in breast milk of Uppsala women (n = 60) 2000-2009 with Swedish women (n = 64) included in the WHO 1989 study**[4]

	**Uppsala 2000-2009**			**WHO 1989**		
	**Median**	**Min**	**Max**	**Median**	**Min**	**Max**
Macroelements						
Ca (mg/L)	307	196	416	235	187	324
K (mg/L)	636	549	729	548	397	711
Mg (mg/L)	28	21	43	34	20	60
Na (mg/L)	192	136	480	88	37	306
P (mg/L)	171	126	233	142	131	150
Microelements						
Co (μg/L)	0.047	0.022	0.38	0.27	0.10	0.75
Cr (μg/L)	0.23	0.026	1.5	1.5	0.61	4.3
Cu (μg/L)	471	327	670	186	80	408
Fe (μg/L)	309	135	794	446	205	1049
Mn (μg/L)	2.6	0.79	8.4	3.2	1.5	26
Mo (μg/L)	2.4	0.80	12	0.40	0.00	5.9
Se (μg/L)	12	8.8	18	13	3.7	25
Zn (μg/L)	3524	1238	5710	700	270	1990
Toxic elements						
As (μg/L)	0.33	0.041	4.6	0.55	0.37	5.5
Cd (μg/L)	0.075	0.028	0.27	0.10	0.1	3.8
Pb (μg/L)	1.2	0.74	6.4	17	9.6	37
Sb (μg/L)	0.031	0.018	0.15	3.0	0.3	23
V (μg/L)	0.039	0.015	0.56	0.13	0.05	0.16

## Discussion

The mammary gland is capable of regulating concentrations of essential elements such as Cu, Fe and Zn in milk to protect the newborn infant against deficiency and excess of these elements [16]. Our knowledge regarding most element concentrations, especially those of toxic metals, in breast milk, and how they are regulated, interact, or affected by maternal exposure, is however, limited. Despite many important advances in analytical techniques for trace element analyses in biological materials, such analyses are still commonly subject to significant errors due to mass interferences, high risk of contamination, and lack of suitable reference materials. Comparisons of breast milk concentration of elements across studies are hampered by differences in analytical performance, state of lactation and factors related to dietary habits and environmental concentrations. Therefore, literature data are inconsistent for many elements, and it is often not possible to conclude whether the differences are real (representing biological or dietary pattern variability) or artifacts arising from analytical difficulties. This is to our knowledge the first study investigating a wide variety of element concentrations in first time, healthy mother’s breast milk, using a highly sensitive analytical method which enables determination of very low concentrations of elements with high precision.

### Macroelements (Ca, K, Mg, Na, P, S)

Comparing our results with those of other studies, in which breast milk samples also were collected during early lactation (in days 11-20, 14-21 and 8-10, respectively) in e.g. Japan [10], Canada [6] and China [8], show striking similarity in concentrations of Ca, K, Mg and P, in spite of quite different dietary patterns. Average concentrations of Ca varied between 274 and 305 mg/L, K between 620 and 639 mg/L, Mg between 26 and 29 mg/L, and P between 170 and 176 mg/L. In poorly nourished Bangladeshi women only, the Ca concentrations (average 261 mg/L at 2 month postpartum) where somewhat lower [17]. Thus, maternal nutritional status seems to influence breast milk concentrations of macronutrients to a limited extent, supporting a tight regulation of these elements [18,19].

The observed differences in median macroelement concentrations between the samples collected in the present study (third week of lactation) and those collected in the 1980’s (third month of lactation) (Table 2) may partly be related to lactation stage, since macroelement concentrations are reported to decrease and stabilize over time [4]. However, the Na concentration in the present study (median 192 mg/L) was twice as high as in the 1989 WHO study (88 mg/L), but comparable to Na concentrations measured in milk samples collected during early lactation in the USA (mean 212 mg/L 21 days pp; [20]) and Gambia (120 mg/L 30 days pp; [21]). Although, breast milk Na concentration is reported to decrease during the lactation period [10,21], the observed difference over time may reflect a true increase in Na intake, since there has been a substantial increase in the intake of salt through food among Swedes since the 1980’s [22]. This needs further investigation since high Na intake in infancy may have long-term effects on blood pressure later in life [21].

### Microelements (Co, Cr, Cu, Fe, Mn, Mo, Se, Zn)

The inter-individual variations in concentrations of microelements were large compared to those for the macroelements, except for Cu and Se. Maternal intake of Cu does not seem to influence milk Cu concentrations [19]. Thus, it seems reasonable that Cu concentration in human milk is regulated. Average concentration of Cu in the present study (mean 471 μg/L) was similar to other results reported from Canada, China, Greece and Japan, ranging from 390 to 480 μg Cu/L [6,8,10,11]. The twice as high Cu concentration in the present study compared to the one in the WHO 1989 study may partly be attributed to a general decline in Cu concentration with lactation stage [5,6], but also to differences in analytical techniques [23].

In contrast to many other essential elements, maternal Se status is reported to influence breast milk Se content [18,24]. Median Se concentration in the present study was lower (12 μg/L) compared to the worldwide median (18 μg/L) at the same stage of lactation [24] and similar to the concentration measured in the 1989 WHO study [4]. The Se intake in Swedish women is relatively low, on average 40 μg/day [25], which is below the recommended intake for lactating women of 60 μg/day (as proposed in the 5th edition of the revised Nordic Nutrition recommendations, NNR 2012, to be published in 2013) or 70 μg/day as proposed by IOM [26]. Fish is an important dietary source of Se, but there was no correlation between Se in breast milk and fish consumption in the present study. We have previously analyzed Se in serum of Swedish non-fish eating women. The median Se concentration was 76 μg/L (range, 53-103 μg/L), which was in agreement with previous studies of the Swedish general population (75 μg/L), indicating that the Se status of non-fish-eating individuals was not substantially lower than that of people who include fish in their diet [27]. A low dietary Se intake in Sweden has been attributed to the poor content of Se in Swedish soils [28]. The low dietary Se intake in Swedish women might require supplementation and warrant further investigations.

Both Zn and Fe concentrations in breast milk are regulated by the mammary gland [19], and decrease with the stage of lactation [5,19,29]. The average Zn concentration in the present study (3.5 mg/L) was similar to those in several other populations at the same stage of lactation [6,10,11], but 5 times higher than the concentration reported in 1989 [4]. The difference may to a large extent be attributed to the substantial decline of Zn, about 50%, during the first 4 months of lactation [10,29]. Zinc deficiency is uncommon in Swedish women, and the Zn level in milk (3.5 mg/L) is adequate to meet the need of breastfed infants for the first several months of life [30,31]. Milk Fe concentrations (median 309 μg/L) in the present study were similar to those reported in other recent studies [11,32], but lower than that reported in 1989 (median 446 μg/L), probably related to the previously reported analytical problems for Fe [4]. Reported average concentrations of Mn in early lactation (days 10-20) vary markedly, from 3.1 to 25 μg/L, with large inter-individual variation [6,10,11] and the present results (3.0 μg/L) were at the lower concentration end. Probably, Mn is mainly transferred by the iron transporters in the mammary gland [17], but the reported wide variation indicates that there may be other transport mechanisms for Mn as well.

Concentrations of Co (median 0.05 μg/L) and Cr (median 0.2 μg/L) were low in the present study compared to the 1989 report (about 15% of 1989 concentration). Some concern about the analytical quality for Cr was raised in the WHO report, as well as in the following publication by Parr et al. [4,23]. Much higher median Cr (25 μg/L) and Co concentrations (0.2-1.5 μg/L) than in the present study were also reported more recently, using modern, sensitive analytical techniques [6,32] with large inter-individual variations, especially for Cr. It is not clear if the large differences in Co and Cr concentrations between individuals and across studies are the results of analytical difficulties or a lack of regulation of those elements in breast milk, or both.

Reported median concentrations of Mo in breast milk vary from 0.5 to 2.5 μg/L [6,9], with the median in our study (2.4 μg/L) being in the upper concentration range. Apparently, Mo milk concentrations decrease with the stage of lactation [6], which could explain the relatively high concentrations of Mo found in the present study compared to 1989 data (median 0.40 μg Mo/L).

### Potentially toxic elements (Ag, As, B, Ba, Cd, Cs, Li, Pb, Rb, Sb, Sr, U, V)

Overall, the concentrations of the toxic elements As, Cd, Pb, Sb and V were lower in the present study than in the 1989 data; the largest difference was found for Pb and Sb. For Pb, the reported median concentration in Swedish women was 17 μg/L in 1989 compared to about 1 μg/L in the present study. Most likely, this decline is mainly related to the Swedish ban of leaded petrol in the 1990’s, which has resulted in significantly decreased blood Pb levels in both mothers and children [33]. For Sb, the difference can probably be attributed to analytical difficulties in the past. For As, we found a positive correlation between concentrations in breast milk and fish consumption, indicating that dietary intake of organic As compounds influences the breast milk concentrations. Furthermore, we could not detect any inorganic As or its methylated metabolites in the breast milk samples with highest concentrations of total As, indicating no or low transfer of inorganic As, the more toxic form of As compounds, to breast milk. We have previously measured mercury in breast milk of Swedish women [34], and found that total mercury in breast milk (0.14 μg/L) was associated with inorganic mercury from amalgam fillings of the mother and not with their fish consumption, i.e. organic methylmercury, in contrast to As. There is a need for identification of the As compounds that passes over to milk in order to evaluate potential consequences for infant health.

We found a negative correlation between Cd and Ca concentrations in breast milk. We have previously suggested that Cd interacts with the transport of essential micronutrients in the mammary gland and that Cd may share common transporters with Fe and Mn for transfer to breast milk, but inhibit secretion of Ca to breast milk [17]. Our present data support that suggestion, despite that Cd concentrations in breast milk were relatively low (median 0.07 μg/L).

The B concentrations in Swedish women (mean 24 μg/L) were similar to B concentrations found in US women (mean about 30 μg/L [29,35]. The B concentration in breast milk was reported not to change during the course of lactation and it was suggested that milk B concentrations are controlled via homeostasis in the mammary gland [29]. However, much higher B concentrations in breast milk (geometric mean about 250 μg/L) were reported from areas in Chile and Argentina where highly elevated B concentrations in drinking water (range 5-10 mg/L) is prevalent [36]. Based on the fact that breast milk B concentrations were 60-100% of plasma B concentrations, it was concluded that B is relatively freely transported from plasma to milk, [36]. The positive correlation of B concentrations in breast milk and fish consumption found in the present study also contradicts a tight regulation of breast milk B. Fish may be a source of B intake since B concentrations in seafood was reported to be higher than in many other foods [37].

The low variation between women in Cs, Rb and Sr breast milk concentrations, together with similar concentrations reported elsewhere (median 2.0, 690 and 31 μg/L, respectively [6], indicates little influence from maternal intake or environmental exposure. There are few data reported for Li in breast milk [36], which varied moderately in our samples, 0.79 to 2.8 μg/L. Also, little is known about Ag and U concentrations in breast milk, which varied largely in our samples, indicating some influence of maternal exposure to these elements, however the concentrations were low.

## Conclusions

There is a need for reliable breast milk element concentration data for designing infant formulas that provide the same nutrition as breast milk and at the same time do not provide excess amounts of trace and toxic elements. The results of the present study support a tight regulation of macronutrients (Ca, K, Mg and P) and several micronutrients (Cu, Zn and Fe) in breast milk, likely reflecting infant requirements. Comparatively higher levels of Na in breast milk were detected which may result from an increased salt intake over time in the Swedish population. This finding needs further investigations in relation to health consequences for mothers and children. The low breast milk Se concentrations, and a low dietary Se intake in Swedish women, might require supplementation and warrant further investigations. We found low concentrations of the toxic elements Pb, Sb and V, and lower concentrations than was previously reported. Also, As, Cd, U and Ag concentrations were low. Other elements that were measured in this study are not very well studied and need further investigation.

## Abbreviations

BMI: Body mass index; CV: Coefficient of variation; LOD: Limit of detection; pp: Postpartum; rs: Spearman correlation; SD: Standard deviation.

## Competing interests

The authors declare that they have no competing interests’.

## Authors’ contributions

MB and KLB performed the statistical analysis and drafted the manuscript. KLB, MV, SL and MB participated in the design of the study. KLB and MV helped to draft the manuscript. MG and BP carried out the sample preparation and element analyses. SL coordinated the sample collection and handling, and provided the background data. All authors read and approved the final manuscript.

## Supplementary Material

Additional file 1 Spearman correlations coefficients (rs in bold = p<0.01; rs in italics = p<0.05) of elements in breast milk, collected during third week of lactation, of first time healthy Swedish mothers (n=60). Click here for file
