# Supplementary material for: Metals and trace element concentrations in breast milk of first time healthy mothers: a biological monitoring study
Source: Environ Health. 2012 Dec 14;11:92. doi: 10.1186/1476-069X-11-92 (PMC3599153; doi:10.1186/1476-069X-11-92)
Supplement: Additional file 1 — Spearman correlations coefficients (rs in bold = p<0.01; rs in italics = p<0.05) of elements in breast milk, collected during third week of lactation, of first time healthy Swedish mothers (n=60). [file 1476-069X-11-92-S1.docx]

Additional file 1: Spearman correlations coefficients (rs in bold = p<0.01; rs in italics = p<0.05) of elements in breast milk, collected during third week of lactation, of first time healthy Swedish mothers (n=60).
